# Calcineurin-NFAT dynamics correspond to cardiac remodeling during aortic banding and debanding, mimicking aortic valve replacement

**DOI:** 10.3389/fmmed.2022.980717

**Published:** 2022-10-25

**Authors:** Ida G. Lunde, Biljana Skrbic, Ivar Sjaastad, Geir Christensen, Cathrine R. Carlson, Theis Tønnessen

**Affiliations:** ^1^ Institute for Experimental Medical Research, Oslo University Hospital and University of Oslo, Oslo, Norway; ^2^ KG Jebsen Cardiac Research Center, University of Oslo, Oslo, Norway; ^3^ Division of Diagnostics and Technology, Akershus University Hospital, Lørenskog, Norway; ^4^ Department of Cardiothoracic Surgery, Oslo University Hospital Ullevaal, Oslo, Norway

**Keywords:** hypertrophy, aortic stenosis, calcium, Rcan gene, fibrosis

## Abstract

Aortic valve stenosis (AS) is a major health problem. Extensive myocardial remodeling increases operative risk and might lead to incomplete reverse remodeling with persistent symptoms after aortic valve replacement (AVR); this makes the optimal timing of AVR a clinical challenge. The pathogenesis behind incomplete reverse remodeling is unclear. Central among signaling pathways in the remodeling heart is the pro-hypertrophic Ca^2+^-activated calcineurin and its downstream nuclear factor of activated T-cell (NFATc1-c4) transcription factors. We investigated calcineurin-NFATc dynamics in patient and mouse hearts during remodeling and reverse remodeling. Myocardial biopsies were obtained from AS patients during AVR and left ventricles harvested from mice subjected to aortic banding (AB) and debanding (DB). The transcript and protein of the NFATc-responsive gene regulator of calcineurin 1-4 (RCAN1-4) and luciferase activity in NFAT-luciferase mice were used as read-outs for calcineurin-NFATc activity. Calcineurin-NFATc activation was sustained through AB 24 h to 18 weeks and elevated in AS patients. All four NFATc isoforms were elevated in AS, while NFATc4 was persistently elevated during chronic remodeling after AB in mice. NFAT activation remained reversible when 1 week’s AB was followed by 1 week's DB and accompanied functional improvement. However, when DB for 1 week followed AB for 4 weeks, NFAT activation was not reversed. In conclusion, calcineurin-NFAT dynamics correspond with cardiac remodeling and reverse remodeling during experimental AB and DB. Our data suggest that calcineurin-NFATc attenuation is important for reverse remodeling and outcomes after AVR for AS.

## Introduction

Myocardial remodeling in response to pathophysiological stimuli is defined by geometric and functional changes of the heart and is a determinant of the clinical course of heart failure (Cohn et al., 2000). Altered gene expression results in molecular, cellular, and interstitial changes where cardiomyocyte hypertrophic growth, inflammation, and excessive collagen accumulation—that is, fibrosis—are central remodeling processes. Reverse remodeling is defined by normative changes of size and function, a capacity of the heart that is demonstrated by advances in medical and device therapies (Kim et al., 2018). Accumulating evidence suggests that the molecular changes associated with remodeling and failure persist in the reverse-remodeled myocardium; nevertheless, more insight is needed to understand the basis of reverse remodeling.

Aortic stenosis (AS) is the most common valvular lesion and a major health problem, with a 2–7% prevalence in the population aged >65 years (Chrysohoou et al., 2011; Rana, 2022). AS causes left ventricular (LV) pressure overload, which elicits myocyte hypertrophy and fibrosis—both of which contribute to left ventricular stiffness (Yarbrough et al., 2012). Hypertrophy is an independent risk factor for symptomatic worsening, including failure (Lloyd-Jones et al., 2002); it is believed that fibrosis occurs somewhat later, coincident with diastolic dysfunction and symptomatic heart failure (Yarbrough et al., 2012). Extensive remodeling leads to increased operative risk during aortic valve replacement (AVR), the treatment for AS, as well as incomplete reverse remodeling with persistent symptoms, morbidity, and mortality after AVR (Lund et al., 2003; Xu et al., 2019). Thus, optimally timing the operation is a clinical challenge. Despite the poor prognosis after the onset of severe symptoms—angina, syncope, and failure (Ross and Braunwald, 1968)—AVR is not performed in 30% of AS patients due to comorbidities that cause high operative risk (Chrysohoou et al., 2011). Insight into the mechanisms underlying remodeling and reverse remodeling could provide the basis for better timing of AVR for AS and of pharmacological treatment for optimal reverse remodeling.

Central among the pro-hypertrophic signaling pathways in the pressure-overloaded myocardium is the Ca^2+^-activated phosphatase calcineurin and its four downstream effectors—the nuclear factor of activated T-cell (NFATc1-c4) transcription factors—all expressed in the heart (Van Rooij et al., 2002; Diedrichs et al., 2004; Molkentin, 2013). Calcineurin dephosphorylates NFATc at serine/threonine residues, leading to nuclear import and NFAT-dependent transcriptional responses, while glycogen synthase kinase 3β (GSK-3β) phosphorylates and inactivates NFATc, thus antagonizing calcineurin (Antos et al., 2002).

Cardiomyocyte Ca^2+^ alterations are central in hypertrophic and failing cardiomyocytes (Balke and Shorofsky, 1998); sustained elevations of intracellular Ca^2+^ activate calcineurin (Molkentin et al., 1998; Molkentin, 2013; Chaklader and Rothermel, 2021). The calcineurin-NFAT pathway is widely accepted as a central director of pathological hypertrophic growth, progressing to heart failure (Wilkins et al., 2004; Berry et al., 2011; Molkentin, 2013).

An estimated 13% of expressed genes in the human heart have NFATc binding sites in their promoter (Putt et al., 2009). Genes having promoters with both NFAT and myocyte enhancer factor 2 (MEF2) sites have the highest odds ratio for differential expression in heart failure (Putt et al., 2009). The regulator of calcineurin 1-4 (RCAN1-4) is a calcineurin-NFATc-responsive gene where all four NFATc isoforms can bind its promotor (Van Rooij et al., 2002; Rothermel et al., 2003). RCAN1-1 and 1-4 are isoforms that include either exon 1 or 4 as alternative first exons, with a dense cluster of 15 NFAT binding sites that regulate exon 4 (Rothermel et al., 2000). The expression of RCAN1-4 is believed to be extremely sensitive to calcineurin-NFATc activity and can be used as an indicator of calcineurin-NFATc activation.

Hence, we investigated activation of calcineurin-NFATc signaling in LV biopsies from AS patients obtained per-operatively during AVR. We paralleled the patient study with a mouse model that mimicked AS and AVR, aortic banding (AB) with subsequent aortic debanding (DB), to study calcineurin-NFATc dynamics during remodeling and reverse remodeling in wild-type (WT) and NFAT-luciferase reporter mice.

## Methods

### Human myocardial tissue samples

The human myocardial biopsy protocol conformed to the Declaration of Helsinki and was approved by the Regional Committee for Research Ethics in Eastern Norway (REK ID 2010/2226). Informed written consent was obtained from all patients. Left ventricular (LV), apical biopsies (5–10 mg) were obtained at Oslo University Hospital, immediately before cross-clamping the aorta in *n* = 17 patients undergoing elective AVR for severe trileaflet AS with preserved ejection fraction (EF >50%) and no significant coronary artery disease (CAD). Myocardial biopsies were also obtained from *n* = 17 patients undergoing elective coronary artery bypass graft (CABG) operations with EF >50%, stable angina pectoris, and no evidence of peri-operative ischemia, previous myocardial infarction, or significant valve disease. Biopsies were taken from the apex of the LV from a normal-appearing and contracting myocardium, and the CABG biopsies served as a control for the AS biopsies. All control patients had a normal ejection fraction on ventriculography and, thus, echocardiography was not routinely performed. Biopsies were taken using a 16G MaxCore Disposable Biopsy Instrument (BARD, GA). Patients were treated according to hospital guidelines. Patient medications and echocardiography measurements were collected from patient records. LV samples were snap-frozen in liquid nitrogen and stored at −70°C until molecular analyses.

### Mouse model of left ventricular pressure overload and its relief

Mouse protocols were reviewed and approved by the Norwegian National Animal Research Committee (IDs 3170 and 6989) and conformed to the NIH Guide for the Care and Use of Laboratory Animals and the ARRIVE guidelines for reporting animal research. Banding of the ascending aorta for 24 h or up to 18 weeks was performed in 7–8-week-old male WT C57BL/6JBomTac mice (Taconic, Skensved, Denmark) as part of a previously published study (*n* = 87) (Melleby et al., 2016). Sham AB consisted of a ligature without tightening. FVB/J NFAT-luciferase reporter mice (Wilkins et al., 2004), kindly provided by Dr. Jeffery D. Molkentin (Cincinnati Children`s Hospital Medical Center, Cincinnati, OH), carry nine copies of NFAT binding sites from the interleukin-4 promoter, upstream of the luciferase gene. AB, sham AB, DB, and sham DB were performed in male WT (*n* = 17) or NFAT-luciferase mice (*n* = 47), as described by Bjørnstad et al. (2012). During sham DB, the loose ligature was removed. All surgery was performed on intubated mice, anesthetized with oxygen and isoflurane (3%). Animals received post-operative analgesia by subcutaneous injection of 0.02 ml Temgesic (0.3 mg/ml). Echocardiography and Doppler flow measurements were performed using a VEVO 2100 (VisualSonics, Canada) in lightly anesthetized mice, to include mice with gradients across the banding site ≥4 m/s 24 h after AB, to re-confirm stenosis before DB, to confirm no stenosis after DB, and before sacrifice. After DB, a normalized aortic flow pattern was confirmed by Doppler echocardiography, as described by Bjørnstad et al. (2012). Body weight was measured, and LV weight (LVW), lung weight (LW), and tibia bone length (TL) were obtained post-mortem. The hearts were rapidly excised, rinsed in PBS, and blotted dry before snap-freezing in liquid nitrogen and storage at -70°C. No tissue was prepared for histology. WT LVs were divided for mRNA and protein analyses, while the entire LV of NFAT-luciferase mice was used for luciferase activity quantification.

### Culturing and cyclic mechanical stress of cardiomyocytes

Primary cardiomyocyte cultures were prepared from heterozygous 1–3 days old NFAT-luciferase mice, as described by Lunde et al. (2011) and Melleby et al. (2016). Cardiomyocytes were plated on collagen I-coated silicone membranes at a density of 2.5*10^5^ cells/ml and kept in a 37°C/5% CO_2_ humidified incubator. The cyclic mechanical stretch (10%, 1 Hz) of cells was induced for 24 h using the FlexCell tension System Fx-4000 (Dunn Labortechnik, Germany). Following 24 h of mechanical stretch, cells were allowed to recover for 24 h. Cells from *n* = 3 separate cell isolations were used, with *n* = 6 technical replicates for each round (total *n* = 18).

### Quantification of luciferase activity

Entire frozen LVs from NFAT-luciferase mice were homogenized with a Polytron 1200 twice for 30 s according to the Luciferase Assay System protocol (Promega, WI). Samples were kept on ice, then vortexed and centrifuged at 12,000 g for 30 s. Luciferase activity in neonatal cardiomyocytes was measured as described (Lunde et al., 2011). Luminescence from duplicates was quantified on a Victor 3 1420 Multilabel Counter (PerkinElmer, MA).

### Transfection of HEK293 cells

For NFATc antibody validation building on our previous report (Lunde et al., 2011), human embryonic kidney 293 (HEK293) cells were cultured and transfected using Lipofectamine 2000 (Invitrogen, NY) according to protocol. The DNA constructs consisted of NFATc1-c4 proteins (NP_940,821, NP_035029, NP_035031, and NP_076188, respectively) cloned in frame with enhanced green fluorescent protein (EGFP) into pEGFP-N1 (Clontech, CA) (custom-made by Genscript Corporation, NJ). Protein was harvested 24 h after transfection. Cells from *n* = 3 separate cell isolations were used, with *n* = 1 technical replicates for each round.

### RNA isolation for real-time RT-PCR analysis

RNA was isolated from frozen LV using the RNeasy mini kit (Qiagen Nordic, Norway). RNA quality, determined using the 2100 Bioanalyzer (Agilent Technologies, CA), was acceptable with RNA Integrity Number (RIN) > 7. Reverse transcription (RT) was performed using the iScript cDNA Synthesis Kit (Bio-Rad). Pre-designed TaqMan assays (Applied Biosystems, CA) were used to determine gene expression on a 7900HT Fast Real Time PCR System and data were analyzed using Sequence Detection Software 2.3 (Applied Biosystems). The following pre-designed TaqMan assays (Applied Biosystems, CA) were used to determine gene expression levels in human tissue—*RCAN1-4* (Hs01120954_m1), *ACTA1* (Hs00559403_m1), *NPPB* (Hs00173590_m1), and *NPPA* (Hs00383230_g1)—and mouse tissue—*Nfatc1* (Mm00479445_m1), *Nfatc2* (Mm00477776_m1), *Nfatc3* (Mm01249194_m1), *Nfatc4* (Mm00452373_g1), *Rcan1-4* (Mm01213406_m1), *Acta1* (Mm00808218_g1), *Nppb* (Mm00435304_g1), *Nppa* (Mm01255747_g1), *Col1a2* (Mm00483888_m1), and *Col3a1* (Mm00802331_m1). *Rpl32* was used for normalization in human (custom-made for *RPL32* exon 32) (Melleby et al., 2016) and mouse (Mm02528467_g1) heart samples. In detail, levels of *RPL32* in AS in the human biopsies were, on average, 0.98-fold that of the controls (statistical testing with *t*-test: non-significant (ns), *n* = 9). In the mouse dataset, *Rpl32* levels were 1.62-fold increased at 24 h post-AB vs. sham (*p* < 0.001), 0.92-fold at AB1w vs. sham (ns), 1.09-fold at AB3w vs. sham (ns), 1.02-fold at AB16w vs. sham (ns), and 0.09-fold at AB18w vs. sham (ns), *n* = 87, and 1.02-fold in AB1wDB1w vs. sham AB1wDB1 (ns), n = 17.

### Immunoblot analysis

Frozen LV tissue and HEK293 cells were supplied in an ice-cold PBS-based lysis buffer containing 1% Triton X-100 (Sigma, MI), 0.1% Tween-20 (Sigma), protease and phosphatase inhibitors (Complete EDTA-free and PhosStop tablets, Roche Diagnostics, Germany, respectively). LVs were homogenized twice for 30 s with a Polytron 1200, while cells were vortexed, left on ice for 30 min, and centrifuged at 20,000 g for 10 min at 4°C. Supernatants were stored at −70°C. SDS-PAGE and immunoblotting was performed according to the Criterion Bio-Rad (CA) protocol using 10–20 μg protein (Lunde et al., 2011). Blots were blocked in 8% dry milk (Bio-Rad), 5% bovine serum albumin (BSA) (Bio-Rad), or 1x casein (Roche Diagnostics) overnight and incubated with antibodies diluted in 2% dry-milk/5% BSA/1x casein for 1 hour. For immunodetection of human NFAT, c1-c4, sc-13033, sc-136206, sc-8321, and sc-13036 antibodies from Santa Cruz Biotechnology (CA) were used, respectively (Lunde et al., 2011). Phospho-NFATc1-c4 were detected using sc-32979 (Ser 259), sc-32994 (Ser 326), sc-32982 (Ser 265), and sc-32630 (Ser 168/170) from Santa Cruz Biotechnology, respectively (Lunde et al., 2011). The theoretical sizes of known NFATc proteins are >90 kDa (Lunde et al., 2011). For detection of RCAN1-1 and RCAN1-4 splice variant proteins, the D6694 antibody from Sigma (MI) was used. For detection of calcineurin, anti-CnAβ (sc-6124, Santa Cruz Biotechnology) was used, while for detection of GSK-3β, antibody #9315 from Cell Signaling Technology was used. Anti-glyceraldehyde 3-phosphate dehydrogenase (GAPDH; sc-20357, Santa Cruz Biotechnology) was used to control loading in human biopsies and HEK293 cells. Anti-vinculin (V9131, Sigma, MI) was used for loading control in mouse hearts. Horse radish peroxidase (HRP)-conjugated secondary antibodies from Southern Biotechnology (#1031, 4030, 6160, AL) were applied to all blots, which were then developed using the ECL Plus Western Blotting Detection System (GE Healthcare, United Kingdom) and Las-4000 mini (Fujifilm, Japan). The blots were stripped (Restore Western Blot Stripping Buffer, Thermo Scientific, Germany) and re-probed. Data processing was performed using ImageJ (NIH, MD) and Adobe Photoshop 2022 (23.3.1).

### Statistics

Data are expressed as scatter plots or group means ± standard error of the mean (SEM). Statistical differences were tested in GraphPad Prism 9 and considered significant for *p* < 0.05. Pearson regression analysis and Pearson linear regression analysis were used for correlations. Unpaired *t*-test or Mann-Whitney test were used when comparing two groups, while one-way ANOVA with Dunnett post-testing was employed for more than two groups.

## Results

### NFATc activation in the myocardium of AS patients

**FIGURE 1:**
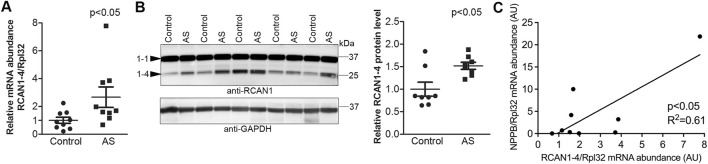
Increased activation of calcineurin-NFAT signaling in the myocardium of aortic stenosis patients. Transmural left ventricular biopsies were collected per-operatively from patients with aortic stenosis (AS) and controls. For patient details, see Table 1. Expression of regulator of calcineurin 1-4 (RCAN1-4) is directly regulated by calcineurin-NFATc activation. **(A)** Relative level of RCAN1-4 mRNA (n = 9). Ribosomal protein L32 (RPL32) was used for normalization. **(B)** Representative immunoblot and quantitative data of RCAN1-4 (*n* = 8). GAPDH was used for loading control. Data shown as single data points and mean ± SEM. Statistical differences were tested using a *t*-test vs. controls. **(C)** Pearson correlation of RCAN1-4 mRNA vs. NPPB mRNA (the transcript encoding brain natriuretic peptide (BNP)), normalized to RPL32, in AS biopsies (*n* = 9).

Transmural biopsies were obtained from the LV of symptomatic AS patients (age, 66.7 ± 3.6 years) with aortic valve areas of 0.68 ± 0.04 cm^2^ and mean aortic gradients of 58.4 ± 4.9 mmHg. All had mild to moderate LV hypertrophy (Lang et al., 2005) (diastolic interventricular septum and posterior wall thicknesses were 1.27 ± 0.04 and 1.23 ± 0.05 cm, respectively), preserved EF (all >50%), and normal LV internal dimensions (4.8 ± 0.2 cm). Consistent with hypertrophic remodeling, the LV expression of α-skeletal actin (encoded by *ACTA1*) was 2.5-fold higher in AS vs. controls (Table 1). LV transcript and protein levels RCAN1-4 were elevated 2.7-fold and 1.5-fold, respectively, in AS biopsies compared to the controls (Figures 1A,B). The RCAN1-4 transcript levels in AS correlated (Figure 1C) with those of heart-failure signature-molecule brain natriuretic peptide (BNP, encoded by *NPPB*), indicating that NFAT activation was related to severity of LV stress. Together, these data suggest that the calcineurin-NFAT pathway was activated in the myocardium of AS patients.

**TABLE 1:** Characteristics of aortic stenosis patients and controls.

	Control patients	AS patients
No. of patients (females)	17 (3)	17 (6)
Years of age at myocardial tissue biopsy	65.5 ± 1.7	66.7 ± 3.6
*Echocardiography*		
Ejection fraction	All >50%	All >50%
Aortic valve area (cm^2^)	NA	0.68 ± 0.04
Max. aortic gradient (mmHg)	NA	90.6 ± 7.7
Mean aortic gradient (mmHg)	NA	58.4 ± 4.9
LVIDd (cm)	NA	4.8 ± 0.2
LVIDs (cm)	NA	2.9 ± 0.2
IVSd (cm)	NA	1.27 ± 0.04
LVPWd (cm)	NA	1.23 ± 0.05
*mRNA expression*		
N	8–9	8–9
*NPPA/RPL32*	1.00 ± 0.62	2.44 ± 1.09
*NPPB/RPL32*	1.00 ± 0.51	4.52 ± 2.43
*ACTA1/RPL32*	1.00 ± 0.19	2.46 ± 0.49*

Epidemiological and echocardiography data (mean ± SEM) of aortic stenosis (AS) and control patients from whom transmural left ventricular (LV) biopsies (5–10 mg) were taken per-operatively. All control patients had normal ejection fraction on ventriculography and, thus, echocardiography was not routinely performed. NA, not available; LVIDd/s, left ventricular internal diameter in diastole and systole, respectively; IVSd, interventricular septal thickness in diastole; LVPWd, left ventricular posterior wall thickness in diastole. Relative mRNA, expression of the hypertrophic marker gene α-skeletal actin (*ACTA1*), and myocardial stress/heart failure markers atrial and brain natriuretic peptides (ANP and BNP encoded by *NPPA* and *NPPB*, respectively), normalized to ribosomal protein L32 (*RPL32*). Drug medications of patients consisted of statins (7 AS/16 controls), acetylsalicylic acid (6 AS/15 controls), β-adrenergic blocker/Ca^2+^ channel antagonist/ACE-inhibitor or AT_1_-antagonist (8 AS/13 controls), bronchodilator (1 control), TNF-α, blocker (1 AS), prednisolone (1 AS), methotrexate (1 AS), folic acid supp. (1 AS), selective serotonin reuptake inhibitors (SSRI, 1 control), proton pump inhibitor (1 AS/1 control), SERM/estrogen supp. (2 AS), α-adrenergic blocker/anti-androgen (2 AS/2 controls), per-oral anti-diabetic (2 controls), Marevan (2 AS), thyroxin supp. (1 AS), and nitroglycerin (1 AS/2 controls). Statistical differences were tested using an unpaired *t*-test; *p < 0.05 vs. control.

### All four NFATc isoforms are up-regulated in the myocardium of AS patients

**FIGURE 2:**
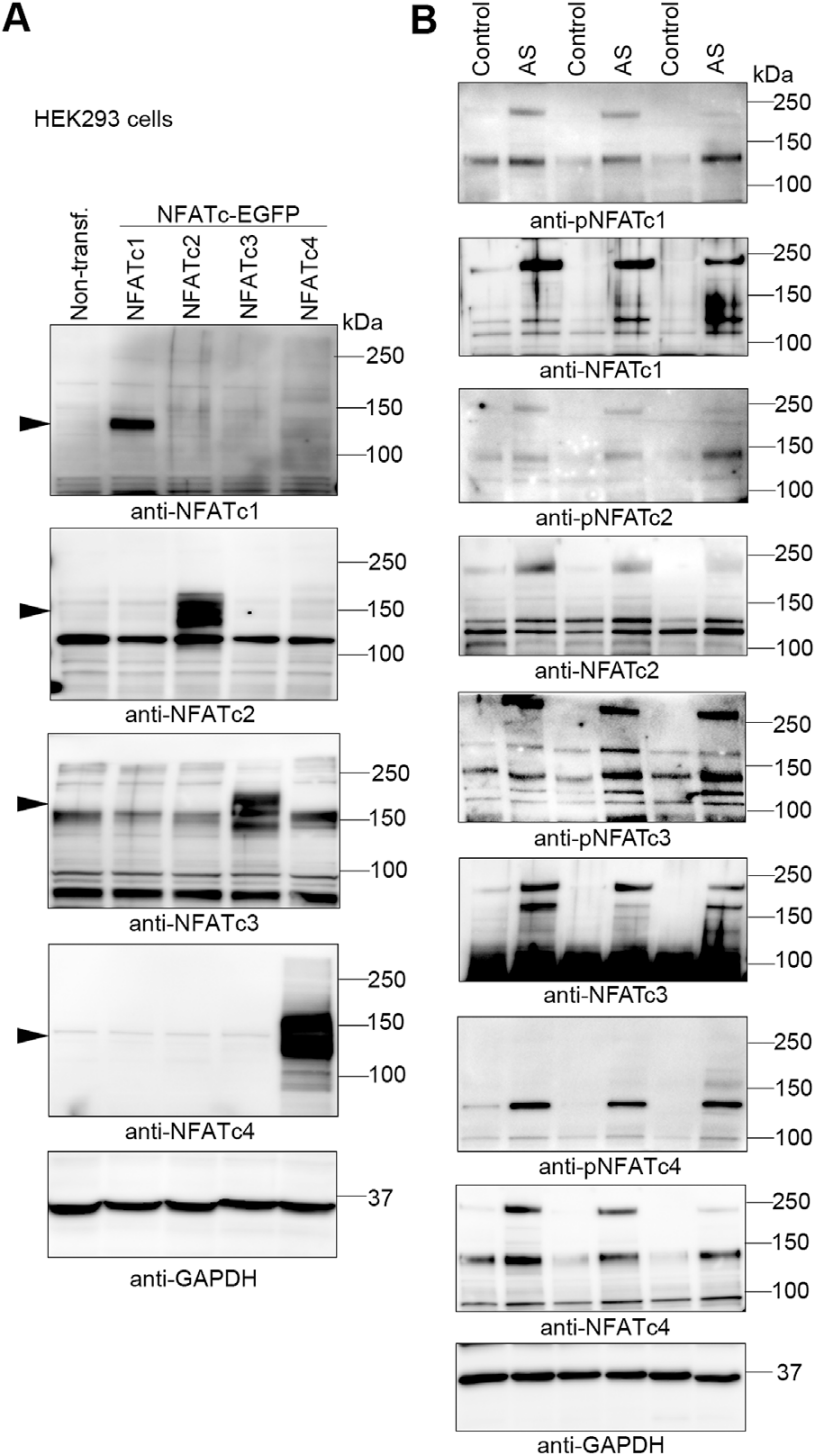
Increased abundance of NFATc1-c4 proteins in the myocardium of aortic stenosis patients. Transmural left ventricular biopsies were collected per-operatively from patients with aortic stenosis (AS) and controls. For patient details, see Table 1. **(A)** Representative immunoblots of nuclear factor of activated T-cell (NFAT) c1, NFATc2, NFATc3, and NFATc4 proteins from NFATc1-c4-transfected human embryonic kidney (HEK) 293 cells. DNA constructs consisted of NFATc1-c4 genes cloned in frame with enhanced green fluorescent protein (EGFP) and were used for antibody validation (*n* = 3). Actin was used for loading control. **(B)** Representative immunoblots of protein and phosphorylation levels of NFATc1-c4 in biopsies from patients AS and controls (*n* = 3). GAPDH was used for loading control.

Understanding endogenous NFATc protein regulation has been especially difficult due to low antibody specificity (Van Rooij et al., 2002; Bourajjaj et al., 2008) and alternative splicing resulting in numerous sub-isoforms—for example, as many as 24 sub-isoforms are predicted for c4 (Vihma et al., 2008). Although we had previously confirmed relatively good NFATc isoform antibody specificity, including detection of phosphorylated NFATc1-c4 (Lunde et al., 2011), we now extended these analyses by overexpressing each GFP-tagged NFATc1-c4 protein in HEK293 cells (Figure 2A). We demonstrated that each antibody exhibited no cross-reactivity with the other isoforms. Using these antibodies, we assessed NFATc protein and phosphorylation in the LV of AS patients and controls. LV immunoblots showed that NFATc1-c4 protein abundance was elevated in AS vs. controls, including phosphorylated (inactive) and non-phosphorylated (activated) NFAT (Figure 2B). It is notable that, since both non-phosphorylated and phosphorylated levels were elevated in AS vs. controls, the quantification of phosphorylated-to-total protein ratio to determine activity level was considered unsuitable. Numerous protein bands probably representing sub-isoforms further complicate such quantifications. Thus, although our data did not allow us to quantify NFATc1-c4 activity levels, their substantially elevated levels suggested that all four NFATc isoforms contributed to NFAT activity in the myocardium of AS patients.

### NFATc activation was sustained during myocardial remodeling in a murine model of AS

**FIGURE 3:**
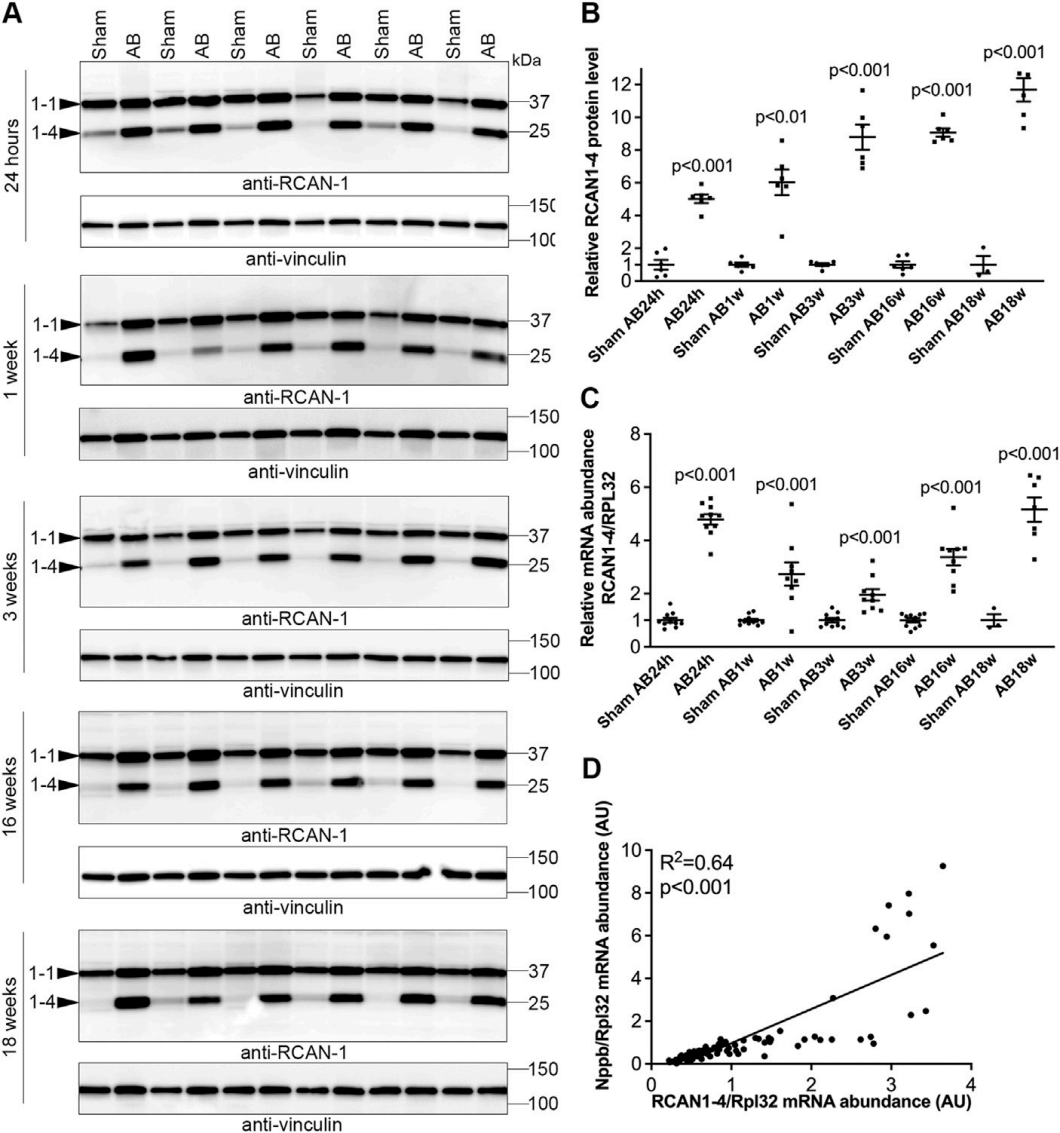
Sustained elevation of cardiac calcineurin-NFATc activation after aortic banding in mice. Wild-type (WT) C57BL/6JBomTac mice were subjected to aortic banding (AB) 24 h–18 weeks as part of a previously published study (Melleby et al., 2016). Controls consisted of sham-operated mice for each time-point. Expression of regulator of calcineurin 1-4 (RCAN1-4) is directly regulated by calcineurin-NFATc activation. **(A)** Representative immunoblots and **(B)** quantitative data of RCAN1-4 in left ventricles (LV) at indicated time-points following AB (*n* = 6). Vinculin was used for loading control. **(C)** Relative mRNA abundance of RCAN1-4 (*n* = 3–10). Ribosomal protein L32 (Rpl32) was used for normalization. Data shown as single data points and mean ± SEM. Statistical differences were tested using *t*-test vs. respective sham-operated controls. **(D)** Pearson correlation of RCAN1-4 mRNA vs. NPPB mRNA (the transcript encoding brain natriuretic peptide (BNP)), normalized to RPL32, in mouse hearts (*n* = 87).

We next investigated NFATc dynamics in an experimental model of AS where WT C57BL/6JBomTac mice were subjected to AB for 24 h over 18 weeks. LVs were harvested as part of a previous study (Melleby et al., 2016). At 24 h post-AB, there was a 5.0-fold increase in RCAN1-4 protein (Figures 3A,B) and a 4.8-fold increase in RCAN1-4 mRNA (Figure 3C) vs. sham-operated mice, consistent with acute NFAT activation in response to pressure overload. Since the housekeeping gene *Rpl32* was increased at AB24hrs, we noted that the increase in RCAN1-4 mRNA was underestimated by normalization; without normalization, RCAN1-4 mRNA was increased 7.8-fold (*p* < 0.001, data not shown). One to three weeks post-AB, at which time there was LV hypertrophy with reduced cardiac function (fractional shortening (FS) ≈15% vs. 20% in sham-operated controls (Melleby et al., 2016)), RCAN1-4 transcripts and protein remained increased (mRNA: 2.7- and 1.9-fold; protein 7.2- and 8.8-fold, respectively; Figures 3A–C). To assess NFAT activation in mice with terminal heart failure due to pressure overload, we studied mice 16–18 weeks post-AB. At these time points, mice had marked LV dilatation and profoundly reduced LV function (FS ≈ 7% (Melleby et al., 2016)), and RCAN1-4 mRNA and protein remained elevated (mRNA: 3.4- and 5.2-fold; protein: 9.1- and 11.7-fold, respectively (Figures 3A–C). Thus, RCAN1-4 mRNA increased the most during the acute phase, 24 h post-AB, while protein was up-regulated the most at later remodeling phases. LV levels of RCAN1-4 mRNA correlated to BNP (Figure 3D), suggesting, as in the human heart, that NFAT activation was related to the severity of LV stress. Altogether, during AB-induced cardiac remodeling in mice, we observed sustained NFATc activation in the heart.

### Expression levels of NFATc were altered during remodeling of the murine heart

**FIGURE 4:**
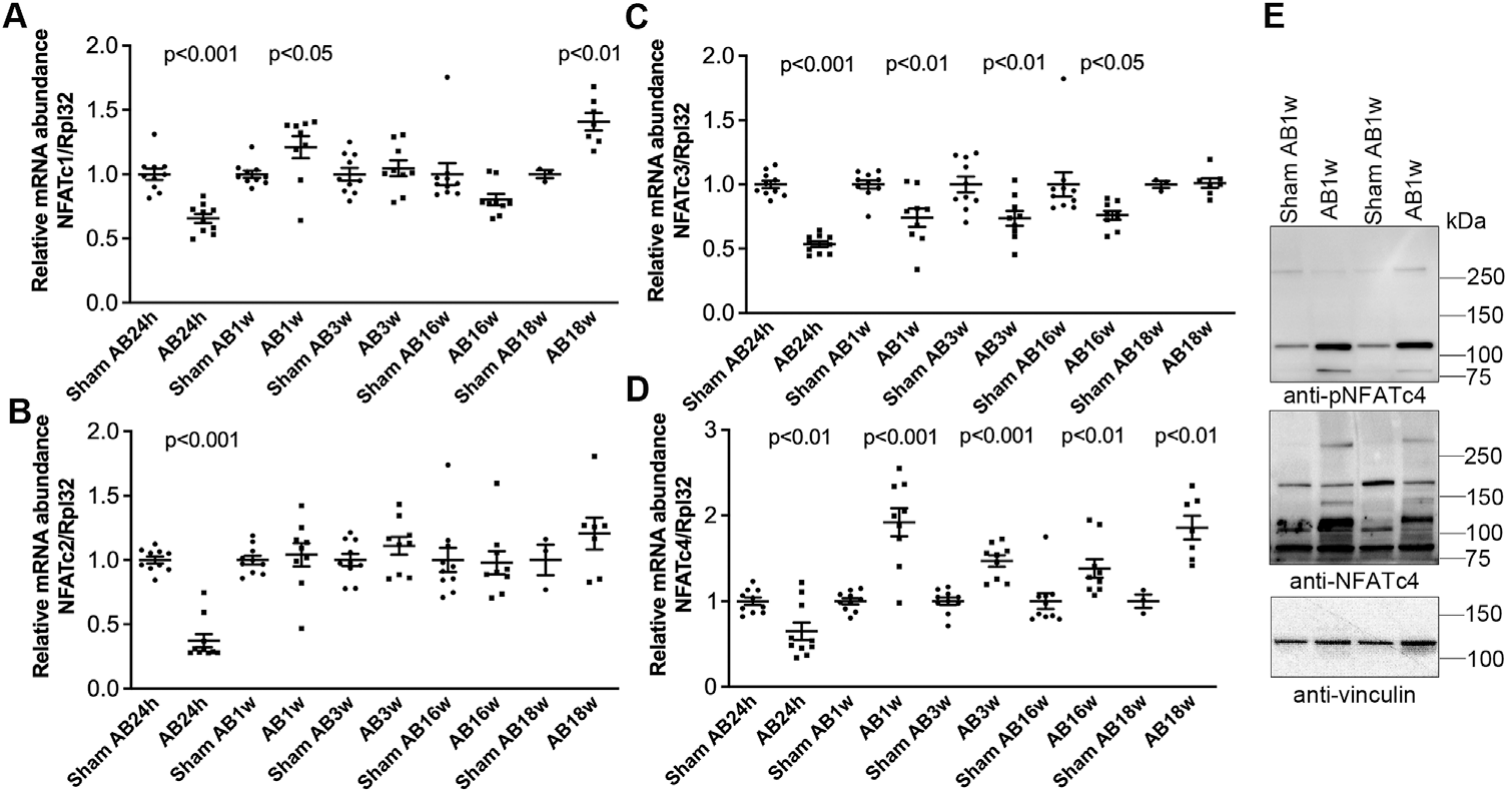
Increased cardiac levels of NFATc4 after aortic banding in mice. Wild-type (WT) C57BL/6JBomTac mice were subjected to aortic banding (AB) 24 h–18 weeks as part of a previously published study (Melleby et al., 2016). **(A–D)** Relative mRNA abundance of nuclear factor of activated T-cell (NFAT) isoforms c1 **(A)**, c2 **(B)**, c3 **(C)**, and c4 **(D)** at indicated time-points following AB, compared to respective sham-operated controls (*n* = 3–10). Ribosomal protein L32 (Rpl32) was used for normalization. Data shown as single data points and mean ± SEM. Statistical differences were tested using *t*-test vs. respective sham-operated controls. **(E)** Representative immunoblot of NFATc4 after AB for 1 week (AB1w) (*n* = 9–10).

To better understand NFATc isoform dynamics in the pressure-overloaded murine heart, we assessed transcript levels after AB or sham in mice. Surprisingly, we observed an initial decrease in all four NFAT isoforms 24 h after AB (Figures 4A–D)—a time point with substantial NFAT activation (Figure 3). However, since the housekeeping gene *Rpl32* was increased at AB24hrs, we re-analyzed NFATc expression without normalization and found that NFATc1 and c4 were unaltered, while NFATc2 and c3 were reduced to 0.58 (*p* < 0.001) and 0.86-fold (*p* < 0.05) that of the control (data not shown). We observed no further alterations in NFATc2 (Figure 4B) at AB1–18 weeks, and a persistent decrease in NFATc3 mRNA throughout cardiac hypertrophy and dilatation (Figure 4C). NFATc1 was increased <1.5-fold at AB1w and AB18w (Figure 4A). NFATc4 mRNA was consistently up-regulated ∼1.5–2.0-fold 1, 3, 16, and 18 weeks after AB (Figure 4D)—during chronic remodeling of the mouse heart—which could imply that NFATc4 particularly contributed to the NFAT activity observed at these time-points. Immunoblotting confirmed increased non-phosphorylated (active) and phosphorylated (inactive) c4 protein after 1 week of AB (Figure 4E). Our data suggested that, in parallel with NFAT activation, the expression levels of NFATc isoforms were altered during remodeling of the mouse heart, with NFATc4 consistently up-regulated during chronic phases.

### Reversal of cardiac NFATc activation and remodeling in a murine model of AVR for AS

Given the sustained NFATc activation in pressure-overloaded human and mouse hearts, we investigated the dynamics of calcineurin-NFAT signaling during reverse remodeling by DB of mice subsequent to AB. DB after 1 week of AB (Table 2) was chosen to mimic the human scenario of AVR to treat AS with preserved EF, as the degree of LV hypertrophy in our biopsy patient cohort (1.5-fold increased LV posterior wall thickness in diastole (LVPWd) vs. normal LVPWd of 0.8 cm (Lang et al., 2005; Table 1) and mice (1.4-fold increased LVPWd; Table 2) were comparable; function (EF and fractional shortening (FS)) was relatively well preserved in both. There was no dilatation at AB1w nor in patients.

**TABLE 2:** Characteristics of wild-type mice subjected to aortic banding and debanding.

	Sham AB1w	AB1w	Sham AB1wDB1w	AB1w DB1w
*Animal and organ weights at harvest*				
N	10	9	11	6
Body weight (g)	27.52 ± 0.45	22.72 ± 0.67***	28.86 ± 0.48	26.62 ± 0.96^###^
LVW/TL (mg/mm)	5.09 ± 0.06	8.25 ± 0.21***	5.28 ± 0.11	5.99 ± 0.12**,^###^
LW/TL (mg/mm)	8.95 ± 0.20	20.01 ± 1.40***	8.52 ± 0.21	10.89 ± 0.92^###^
*Echocardiography*				
N	6	7	5	6
LAD (mm)	1.70 ± 0.02	3.02 ± 0.09***	1.65 ± 0.02	2.60 ± 0.14***^,#^
LVPWd (mm)	0.75 ± 0.03	1.08 ± 0.02***	0.80 ± 0.04	0.96 ± 0.04**^,#^
LVIDd (mm)	4.08 ± 0.11	4.09 ± 0.05	4.16 ± 0.10	4.10 ± 0.14
FS (%)	18.87 ± 1.29	14.29 ± 1.22*	22.92 ± 2.34	17.76 ± 1.24^#^
*LV mRNA expression*				
N	10	9	11	6
*Nppa/Rpl32*	1.00 ± 0.10	10.99 ± 1.65***	1.00 ± 0.08	7.04 ± 1.37**^,##^
*Nppb/Rpl32*	1.00 ± 0.09	2.98 ± 0.43***	1.00 ± 0.05	1.89 ± 0.10*^,#^
*Acta1/Rpl32*	1.00 ± 0.22	6.54 ± 1.26***	1.00 ± 0.15	1.93 ± 0.24^###^
*Col1a2/Rpl32*	1.00 ± 0.07	15.35 ± 1.83***	1.00 ± 0.05	4.95 ± 1.08**^,###^
*Col3a1/Rpl32*	1.00 ± 0.08	13.10 ± 1.63***	1.00 ± 0.06	6.35 ± 1.51**^,###^

Post-mortem organ weights, M-mode echocardiographic recordings and mRNA, expression data of male wild-type C57BL/6JBomTac mice after aortic banding (AB) or sham-operation for 1 week, or 1 week of aortic debanding subsequent to 1 week of aortic banding (DB; AB1wDB1w) and of sham-operated controls. Relative mRNA, expression of skeletal muscle α-actin (*Acta1*), atrial and brain natriuretic peptides (ANP and BNP encoded by *Nppa* and *Nppb*, respectively), collagen I (*Col1a2*), and III (*Col3a1*), normalized to ribosomal protein L32 (*Rpl32*) expression. LVW, left ventricular weight; TL, tibia length; LW, lung weight; LAD, left atrial diameter; LVPWd, left ventricular posterior wall thickness in diastole; LVIDd, left ventricular internal diameter in diastole; FS, fractional shortening. Statistical differences were tested using a one-way ANOVA, with Dunnett’s multiple comparison test vs. sham AB1w; *p < 0.05; **p < 0.01; ***p < 0.001 or vs. AB1w; ^#^ *p* < 0.05,^##^ *p* < 0.01;^###^ *p* < 0.001 vs. AB1w.

**FIGURE 5:**
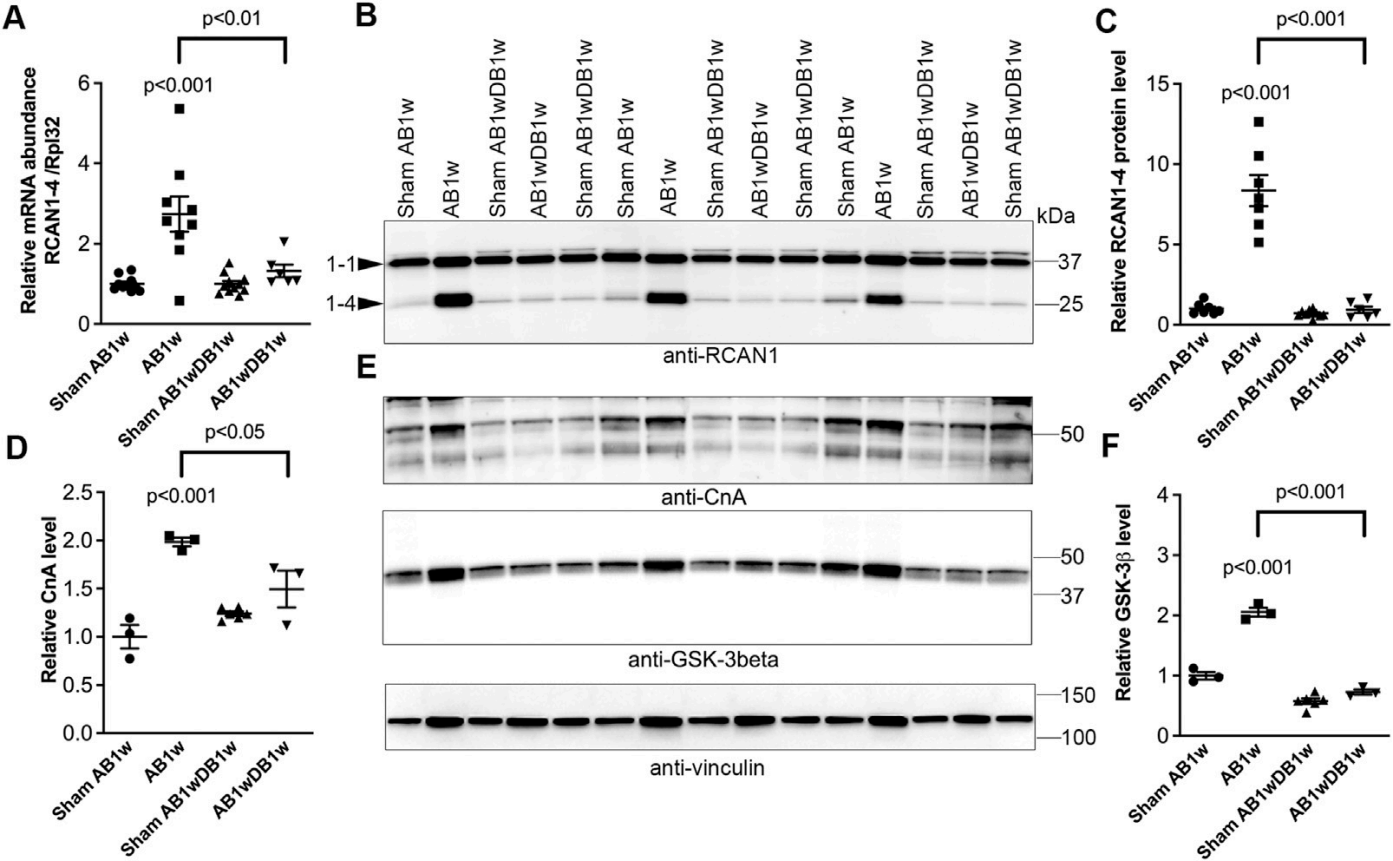
Cardiac calcineurin-NFAT activation induced by aortic banding was reversed by relief of pressure overload in mice. Wild-type (WT) C57BL/6JBomTac mice were subjected to aortic banding (AB) or sham operation for 1 week (AB1w and Sham AB1w, respectively), or 1 week of aortic de-banding subsequent to 1 week of aortic banding (DB; AB1wDB1w) and of sham-operated controls (Sham AB1wDB1w). For animal characteristics, see Table 2. Expression of regulator of calcineurin 1-4 (RCAN1-4) is directly regulated by calcineurin-NFATc activation. Relative mRNA (**(A)**; *n* = 6–11) and protein (**(B,C)**; *n* = 6–10) levels of RCAN1-4. Expression of ribosomal protein L32 (Rpl32) was used for mRNA normalization. **(D,E)** Relative protein levels of calcineurin (CnA subunit). **(E,F)** Relative protein levels of glycogen synthase kinase 3β (GSK-3β). D-F *n* = 3–6. Vinculin was used for protein loading control. Data shown as single data points and mean ± SEM **(A,C,D,F)**. Statistical differences were tested using a one-way ANOVA with Dunnett’s multiple comparison test vs. sham AB1w or AB1w.

To investigate whether DB could reverse NFATc activation, we measured levels of RCAN1-4 transcripts (Figure 5A) and protein (Figures 5B,C) in the LV of AB-DB WT mice (Table 2). Importantly, RCAN1-4 mRNA and protein were normalized and comparable to control levels, implying complete reversal of NFAT activation. In these WT mice, DB reversed hypertrophy—as seen by reduced LVW, LVPWd, and expression of *Acta1*, compared to AB1w, normalized body and lung weight, and FS, and reduced the expression levels of *Nppa* and *Nppb*. Transcripts encoding collagen I (*Col1a2*) and collagen III (*Col3a1*) were also reduced by DB to levels 0.32-fold and 0.48-fold that of AB1w. Correlation analyses in the AB1wDB1w samples showed no association between RCAN1-4 mRNA and *Col1a2* nor *Col3a1* (data not shown, *n* = 6), thus suggesting that mechanisms other than NFAT reversal were responsible for the diminution of pro-fibrotic pathways.

Finally, the protein levels of calcineurin itself (Figures 5D,E) and GSK-3β (Figures 5E,F) roughly doubled upon AB1w and returned to levels found in sham mice, 1 week after DB. These data support NFAT reversal in the LV being associated with reverse remodeling and improvement in LV function.

### Prolonged pressure overload prior to its relief inhibits reversal of NFATc activation and reverse remodeling in a murine model of AVR for AS

The AB of NFAT-luciferase reporter mice for 1 week caused LV hypertrophy as seen from echocardiography, including increased LWPWd, increased left atrial diameter (LAD) and LV internal diameter in diastole (LVIDd), decreased body weight, and increased lung weight (Table 3). Thus, these FVB/J mice had a more severe phenotype after 1 week of AB compared to WT C57BL/6JBomTac mice (Table 2); LV dilatation was already present then. DB after 1 week normalized body weight, improved cardiac function (FS from ≈12% to 18%) and, although cardiac dimensions, LAD, and lung weight remained greater than the sham-operated, they were more improved in debanded than banded mice (Table 3).

**TABLE 3:** Characteristics of NFAT-luciferase mice subjected to aortic banding and debanding.

	Sham AB1w	AB1w	Sham AB1wDB1w<	AB1wDB1w	Sham AB4w	AB4w	AB4wDB1w
*Animal and organ weights*							
N	9	11	8	9	2	4	4
Body W (g)	26.83±0.18	23.83±0.60***	26.24±0.46	26.94±0.54^###^	28.30±0.60	26.50±0.23	27.70±0.26^$^
LVW/TL (mg/mm)	5.24±0.08	8.13±0.14***	5.01±0.18	6.64±0.19***^,###^	5.57±0.31	7.63±0.32	5.44±0.24^$$^
LW/TL (mg/mm)	9.19±0.23	22.00±1.44***	8.91±0.29	13.78±0.45*^,###^	8.98±0.84	18.27±1.77	10.36±0.50^$$^
*Echocardiography*							
N	5	8	5	5	2	4	4
LAD (mm)	1.51±0.04	2.94±0.07***	1.35±0.04	2.31±0.09***^,###^	1.69±0.09	2.58±0.07	2.16±0.22
LVPWd (mm)	0.85±0.02	1.08±0.04***	0.86±0.03	1.02±0.02*	0.83±0.05	0.86±0.07	0.87±0.08
LVIDd (mm)	3.53±0.14	4.52±0.12***	3.84±0.10	4.17±0.11*^,#^	4.00±0.05	4.37±0.15	4.35±0.14
FS (%)	23.6±1.17	12.1±0.98***	25.2±2.07	17.7±2.56*^,#^	25.4±1.01	14.8±2.11	14.3±1.08

Post-mortem and echocardiography data of male FVB/J NFAT-luciferase reporter mice following 1 (AB1w) or 4 (AB4w) weeks of aortic banding (AB), 1 week of AB, and subsequently 1 week of debanding (DB; AB1wDB1w), 4 weeks of AB, with 1 week of DB (AB4wDB1w), and of sham-operated controls. LVW/TL, left ventricular weight/tibia length; LW/TL, lung weight/tibia length. M-mode echocardiography recordings: LAD, left atrial diameter; LVPWd, LV, posterior wall thickness in diastole; LVDd, LV, internal diameter in diastole; FS, fractional shortening. Statistical differences were tested using a one-way ANOVA, with Dunnett’s multiple comparison test vs. sham AB1w; *p < 0.05, ***p < 0.001 or vs. AB1w;^#^ *p* < 0.05,^###^ *p* < 0.001, or a *t*-test comparing AB4wDB1w vs. AB4w; ^$^ *p* < 0.05, ^$$^ *p* < 0.01. No statistical testing was performed vs. Sham AB4w due to *n* = 2.

**FIGURE 6:**
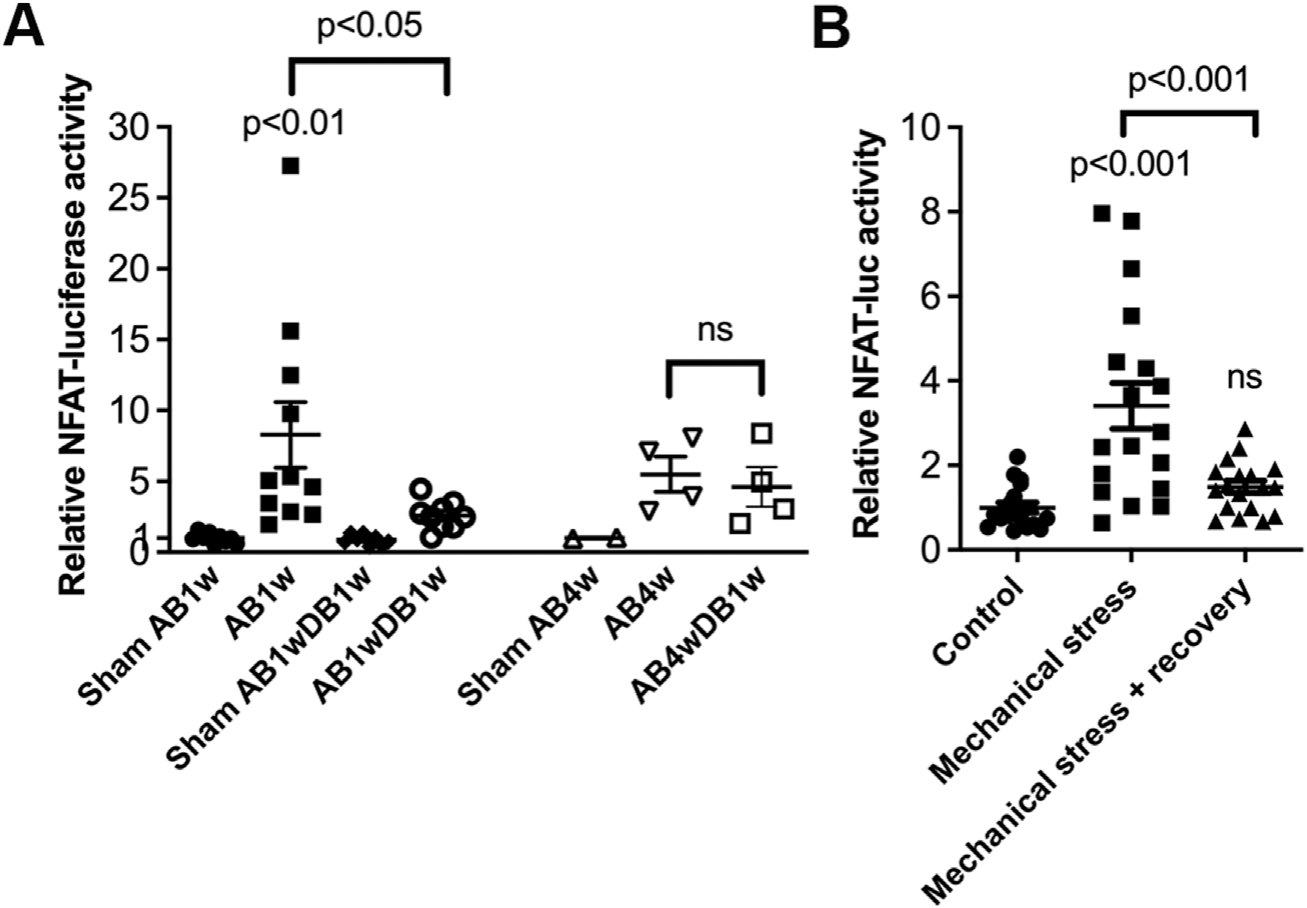
Prolonged pressure overload prior to its relief attenuated reversal of cardiac calcineurin-NFATc activation in mice. NFAT-luciferase reporter mice were subjected to 1 (AB1w) or 4 (AB4w) weeks of aortic banding (AB) or sham-operation, or 1 or 4 weeks of AB followed by 1 week of aortic de-banding (DB; AB1wDB1w or AB4wDB1w). For animal characteristics, see Table 3. Data shown as single data points and mean ± SEM. **(A)** NFAT-luciferase activity was measured in entire left ventricles (LVs). Statistical differences were tested using a one-way ANOVA with Dunnett’s multiple comparison test vs. sham AB1w and vs. AB1w. A *t*-test was used to compare AB4wDB1 vs. AB4w; ns, non-significant. No statistical testing was performed vs. Sham AB4w due to *n* = 2. N = 8–11 for 1w dataset; *n* = 2–4 for 4w dataset. **(B)** NFAT-luciferase activity in cardiomyocytes from neonatal NFAT-luciferase mice following 24 h of mechanical stress, with or without 24 h of recovery. Cells from *n* = 3 separate cell isolations were used, with *n* = 6 technical replicates for each round (total *n* = 18). Statistical differences were tested using one-way ANOVA with Dunnett’s multiple comparison test vs. control cells, or vs. stress.

Entire LVs of NFAT-luciferase reporter mice were analyzed for NFAT-luciferase activity. Banded NFAT-luciferase mice had an 8.3-fold increase in NFAT-luciferase activity at AB1w, which decreased with DB to levels 2.6-fold that of sham (Figure 6A). Next, we prolonged AB to 4 weeks, where the cardiac phenotype had progressed with thinning of the LVPWd (Table 3). Importantly, when AB was prolonged prior to relief of banding for 1 week, we did not observe reversal of NFAT-luciferase activity (5.5-fold vs. 4.6-fold increased, respectively). Although the LV and lung weights had reversed, echocardiography showed no differences in FS or dimensions (Table 3). These data supported the conclusion that NFAT reversal corresponded to reverse remodeling and improvement in LV function and that prolonged pressure overload prior to its relief attenuated reversal of cardiac NFATc activation and functional improvement in mice.

Finally, to assess reversal of NFAT activity in isolated cardiomyocytes, NFAT-luciferase activity was assessed in an *in vitro* model of pressure overload. In neonatal rat cardiomyocytes, NFAT activity increased 3.4-fold by cyclic mechanical stress and reversed after recovery (Figure 6B).

## Discussion

We here show that, in the LV of patients with AS and mice subjected to AB, there is sustained NFATc activation. NFATc activation was measured by levels of the direct NFATc target gene RCAN1-4 and NFAT luciferase activity in reporter mice. In myocardial biopsies taken during AVR from AS patients, we found increased levels of NFATc1-4 proteins, both phosphorylated and non-phosphorylated, suggesting that they all are involved in the downstream remodeling processes. In mouse hearts post-AB, NFATc expression was altered, and NFATc4 was consistently elevated during the chronic remodeling phases, suggesting a particular role. Although NFATc activation was increased in AS and AB, fractions of NFATc1-c4 remained phosphorylated—that is, inactive—suggesting that calcineurin-NFATc antagonizing pathways are active in parallel. In a murine model of AVR where pressure overload was relieved by DB, NFAT activation was reversed *in vivo* and cardiac function improved. Similarly, NFAT activation triggered by mechanical stress was reversed in cardiomyocytes *in vitro* after a recovery phase. Finally, prolonged pressure overload prior to DB inhibited NFAT reversal and functional improvement, suggesting that the timing of relief of pressure overload is important for NFAT inactivation and reverse remodeling of the heart.

Several heart failure therapies that improve outcome are associated with regression of hypertrophy (Groenning et al., 2000; Devereux et al., 2004). Targeting the hypertrophic response itself requires that the responsible signaling pathways remain reversible. Moreover, reverse remodeling has been associated with a gene expression profile different from the mere reversal of pathways activated during remodeling (Stansfield et al., 2009). We here set out to investigate whether the central pro-hypertrophic pathway, calcineurin-NFATc, was reversible upon DB. Since calcineurin-NFATc studies rely heavily on murine models in which genetic manipulations have been initiated before birth, little is known about calcineurin-NFATc reversibility. Interestingly, inducible activation of calcineurin in the cardiomyocytes of adult mice was found to trigger pathological hypertrophy preceding systolic dysfunction, fetal gene activation, fibrosis, and clinical heart failure (Berry et al., 2011). Cardiac hypertrophy and fetal gene expression reversed when calcineurin activity was turned off, while fibrosis only partially reversed. Building on these results, we here show that NFATc activation induced by AB could be attenuated by DB and was accompanied by regression of LV hypertrophy, improved contractile function, attenuated pulmonary congestion and the normalization of body weight. We speculate that, mechanistically, it is likely that reduced mechanical stress is responsible for reversing the Ca^2+^-calcineurin-NFAT axis, affecting NFAT phosphorylation and the protein levels of calcineurin, GSK-3β, and NFAT.

Whether fibrosis remains reversible, or mostly preventable, represents an area of intense research in cardiology, and the DB model is important for future studies. The lack of histology sections in our study represents a limitation that prevents us from reaching a conclusion about the regression of cardiomyocyte size and fibrosis after DB; however, the reductions we found in expression of molecular markers—*Acta1*, *Col1a2*, and *Col3a1*—suggest a diminution of pro-hypertrophic and pro-fibrotic pathways in cardiomyocytes and cardiac fibroblasts, respectively. Although Ca^2+^-calcineurin-NFAT signaling in the heart is mainly described in cardiomyocytes, this axis has been detected in cardiac fibroblasts and is activated upon increased mechanical tension, regulating the myofibroblast phenotype (Lighthouse and Small, 2016). During remodeling and failure, cardiac fibroblasts transdifferentiate into myofibroblasts: proliferative, contractile cells producing excessive amounts of extracellular matrix, including collagens I and III. As we found no correlation between *RCAN1-4* and *Col1a2* nor *Col3a1* expression in ABDB hearts, our findings suggested that mechanisms other than NFAT reversal were responsible for the diminution of pro-fibrotic pathways after DB. Since the NFAT signal in LV tissue likely represents mostly cardiomyocytes, we speculate that the reduction in collagen expression observed was a result of reduced mechanical stress sensed by fibroblasts directly, although it could be secondary to reverse remodeling in cardiomyocytes.

Extensive and prolonged remodeling in AS patients is associated with incomplete reverse remodeling, persistent symptoms, morbidity, and mortality after AVR, making optimal timing of surgery a clinical challenge (Lund et al., 2003). We modeled a delayed AVR in AS by extending the period of AB in NFAT luciferase mice from 1 to 4 weeks before DB, where the remodeling prior to DB had progressed. Although *n* was relatively low due to the challenging nature of the experiments, we were able to show that the reversal of NFATc signaling was dependent on the timing of DB, as we did not observe NFATc reversal. In contrast to the reversal of NFAT activation in mice debanded 1 week after banding, we found no reversal of NFAT activity nor improvement in function when DB occurred after 4 weeks. It is thus likely that some of the deleterious effects of calcineurin-NFATc signaling in AS hearts can be reversed by AVR, with timing of AVR being a crucial factor for the reversal of NFAT-dependent remodeling mechanisms. In this regard, it would be interesting for future research to relate calcineurin-NFATc activation in biopsies—such as RCAN1-4 levels at AVR—to reverse remodeling on follow-up. Furthermore, it is possible that more extended relief of pressure overload, which was not studied here, could lead to more reverse remodeling, both in patients on follow-up and in mice with a longer duration of DB.

Studies in mice have indicated that the pharmacological inhibition of calcineurin using cyclosporine A or FK506 can prevent hypertrophy following pressure overload (Molkentin et al., 1998; Sussman et al., 1998; Molkentin, 2013). Recently, adeno-associated virus (AAV)-mediated expression of NFAT decoy oligonucleotides was shown to offer protection from cardiac hypertrophy and heart failure (Remes et al., 2021). Since we also show here that, during the earlier stages of the remodeling process, calcineurin-NFATc signaling remains reversible, we should perhaps remain optimistic regarding future calcineurin-NFAT inhibition using pharmacotherapy—for instance, in patients with incomplete reverse remodeling and in those with risk factors preventing them from having surgery.

Understanding endogenous NFATc regulation has been difficult, especially due to low antibody specificity (Van Rooij et al., 2002; Bourajjaj et al., 2008) and alternative splicing resulting in numerous sub-isoforms (Vihma et al., 2008). Although initially found in T-cells, most mammalian tissues express one or more of the four NFATc genes, such as cardiomyocytes which express all four. Although there are indications that NFATc4 is activated in hypertrophic and failing human hearts (Diedrichs et al., 2004), little is known about NFATc1-c3. By extending our understanding, our data suggest that all four calcineurin-dependent NFATc isoforms participate in the hypertrophic response of the human heart.

In the mouse heart, the expression of all four isoforms was altered at the mRNA level; however, only NFATc4 was consistently up-regulated during chronic remodeling phases. Both phosphorylated and non-phosphorylated NFATc4 were found elevated. Previous mouse studies have shed light on the specific effects and functional redundancy among NFATc proteins. NFATc1 knock-out is embryonically lethal due to cardiac morphogenesis and valvular defects which resemble common congenital human heart defects (De la Pompa et al., 1998; Ranger et al., 1998). NFATc2 nulls are viable and do not develop calcineurin-dependent hypertrophy and failure (Bourajjaj et al., 2008). Likewise, NFATc3-null mice demonstrate reduced hypertrophy after pressure overload (Wilkins et al., 2002). NFATc4 knock-out mice do not show compromised hypertrophic growth (Van Rooij et al., 2002; Wilkins et al., 2002), although c4 overexpression results in massive cardiac hypertrophy (Molkentin et al., 1998). Angiotensin II and norepinephrine activate specific NFATc isoforms in ventricular cardiomyocytes (Lunde et al., 2011) and that NFATc1 and c3 are differentially regulated in atrial and ventricular myocytes (Rinne et al., 2010). These data suggest some functional specification of NFATc in the remodeling of the heart; however, their differential effects remain elusive as their DNA target site is the same—for example, they all activate the RCAN1-4 promoter (Van Rooij et al., 2002)—and the DNA binding domain is conserved (Hoey et al., 1995; Hogan et al., 2003). More pronounced effects are seen when several NFATc proteins are lacking; however, the generation of mice lacking all four has failed due to lethality (Van Rooij et al., 2002). Mice lacking c3 and c4 show embryonic lethality with a thin ventricle (Bushdid et al., 2003), and the phenotype can be rescued by re-expression of c4, which is in line with some degree of functional redundancy.

We conclude that calcineurin-NFAT dynamics correspond to cardiac remodeling and reverse remodeling during experimental AB and DB. Calcineurin-NFATc activation is a sustained response to pressure overload and its reversal corresponds to reverse remodeling and functional improvement in mice. Our data suggest that calcineurin-NFATc attenuation is important for reverse remodeling and outcomes after AVR for AS.

## Data Availability

The original contributions presented in the study are included in the article further inquiries can be directed to the corresponding author.
